# Oral tegafur-uracil as metronomic therapy following intravenous FOLFOX for stage III colon cancer

**DOI:** 10.1371/journal.pone.0174280

**Published:** 2017-03-22

**Authors:** Wen-Yen Huang, Ching-Liang Ho, Chia-Cheng Lee, Cheng-Wen Hsiao, Chang-Chieh Wu, Shu-Wen Jao, Jen-Fu Yang, Cheng-Hsiang Lo, Jia-Hong Chen

**Affiliations:** 1 Department of Radiation Oncology, Tri-Service General Hospital, National Defense Medical Center, Taipei, Taiwan; 2 Institute of Clinical Medicine, National Yang-Ming University, Taipei, Taiwan; 3 Division of Hematology/Oncology, Department of Medicine, Tri-Service General Hospital, National Defense Medical Center, Taipei, Taiwan; 4 Division of Colon and Rectal Surgery, Department of Surgery, Tri-Service General Hospital, National Defense Medical Center, Taipei, Taiwan; 5 Graduate Institute of Clinical Medicine, College of Medicine, Taipei Medical University; Institut national de la recherche scientifique, CANADA

## Abstract

The purpose of this study was to estimate the impact of metronomic therapy with oral tegafur-uracil (UFUR) following an intravenous FOLFOX regimen as surgical adjuvant chemotherapy on the overall survival (OS) and disease-free survival (DFS) of stage III colon cancer patients. From the retrospective database of patients who underwent a surgical resection for colorectal cancer at the Tri-Service General Hospital from October 2008 through December 2014, stage III colon carcinomas treated with radical R0 resection were reviewed. One hundred thirty two patients were treated with a FOLFOX regimen (comparison group), and 113 patients were treated with the same regimen followed by additional oral UFUR (UFUR group). The clinical characteristics and mean age of the comparison and UFUR groups were similar. Furthermore, for all study patients, DFS was not significantly different between the two groups. However, 5-year OS rates were 86.8% and 68.5% in the UFUR and comparison groups, respectively (p = 0.0107). Adding UFUR to a FOLFOX regimen was found to significantly improve the OS in patients with stage III colon cancer. UFUR as a maintenance therapy following FOLFOX regimen as an alternative therapeutic option for the treatment of stage III colon cancer patients.

## Introduction

Globally, colon cancer is the third most common cancer in men and the second in women. In 2012, there were an estimated 1.4 million new colorectal cancer cases and 693,900 deaths[1,2]; the highest incidence rates are in Japan, Europe, Oceania, and North America. However, the mortality rates are decreasing in many countries worldwide, likely due to screening and improved treatment[3]. According to the 2015 Surveillance, Epidemiology, and End Results database, the 5-year survival rate of patients with colon cancer is 90.1% for those whose disease is confined to the primary site at diagnosis. However, it is decreased to 70.8% when the tumor has spread to the regional lymph nodes. Colon cancers with regional lymph node metastasis are classified as at least stage III disease in the seventh edition of the American Joint Committee on Cancer staging system. Their current standard treatment is colectomy with en bloc resection of regional lymph nodes followed by adjuvant chemotherapy with a FOLFOX or CapeOx regimen. However, the survival rate of these patients is significantly lower than that of patients with node-negative disease. Thus, it is necessary to explore novel drug targets and treatment strategies to improve outcomes. Metronomic chemotherapy, which is continuous low dose anticancer therapy, is a new, emerging concept in cancer treatment that has resulted in favorable outcomes in some cancer patients.

Conventional chemotherapy administration is generally based on the concept of the maximum tolerated dose, which is the dose that maximizes cancer cell death with tolerable injury to normal cells. However, the majority of patients experience tumor regrowth after a period of disease regression or stabilization. Metronomic chemotherapy is maintenance therapy with low doses of cytotoxic drugs that are administered at shorter intervals in consecutive doses and without interruption in order to exert a sustained cytotoxic or apoptotic effect. The potential of metronomic chemotherapy was revealed in animal studies two decades ago, and its safety and efficacy have been investigated in some human cancers, such as cancer of the central nervous system[4,5], breast[6], lung[7], liver[8,9], colon[10,11], prostate[12,13], kidney[14,15], and ovary[16,17], among others. For colon cancer, most previous studies focus on metastatic disease; reports regarding stage III colon cancer are lacking.

Tegafur-uracil (UFUR®; TTY Biopharm Co, Taiwan) is an orally administered fluoropyrimidine inhibitor of dihydropyrimidine dehydrogenase. It is a fluorouracil (FU) derivative chemotherapeutic drug composed of tegafur and uracil in a 1:4 molar ratio. Tegafur is an orally bioavailable prodrug of 5-FU, and uracil reversibly inhibits the primary catabolic enzyme for 5-FU, namely dihydropyrimidine dehydrogenase. Its administration can consistently achieve an adequate plasma FU concentration with a low toxicity rate. Thus, it could be an effective choice for metronomic chemotherapy.

In this study, we aim to compare the clinical results of patients with stage III colon cancer after metronomic chemotherapy with oral tegafur-uracil with those who were not treated with metronomic chemotherapy.

## Materials and methods

This was a retrospective study. From the database of patients who underwent a surgical resection for colon cancer at the Tri-Service General Hospital from January 2008 through January 2015, newly diagnosed patients with stage III colon carcinoma in whom a radical R0 resection was performed were reviewed. The database contained detailed information about patient characteristics, operative findings, histological examination, laboratory findings, and adjuvant therapies. Cancers of the rectum were excluded because their standard treatment modalities are different from those used to treat cancer of other locations of the colon. This study was approved by the ethics review boards of the Tri-Service General Hospital. We verified that patient records were de-identified and analyzed anonymously. A total of 313 patients were identified. Of them, 245 patients underwent postoperative adjuvant chemotherapy with a FOLFOX regimen. No patient received chemotherapy before surgery.

Because the evidence for using oral UFUR in stage III colon cancer is lacking, physicians prescribed the drug according to their personal preference. In the UFUR group, UFUR was administered orally after the completion of a postoperative FOLFOX regimen. The total daily dose was 400 mg divided into two doses after meals for 7 days a week without interruption. The duration of UFUR use was not universal. Because the benefit of metronomic use of UFUR was not yet clinically evident, we had no consensus on the duration of UFUR use. Most physicians prescribed UFUR with total 3–12 months. In the comparison group, patients did not receive anti-cancer treatment after completion of a FOLFOX regimen, except in cases of disease relapse.

The follow-up survival data were collected retrospectively based on analyses of medical records. Cancer was staged using the seventh edition of the American Joint Committee on Cancer staging system. All patients underwent regular follow-up examinations, including serial serum carcinoembryonic antigen measurements every 3 months for at least 3 years and 4–6 months thereafter, and abdominal ultrasonography or computed tomography, chest radiography, and colonoscopy every 12 months. However, image surveys were conducted immediately for patients in whom disease progression or relapse were suspected.

Age is presented as the mean, median, and range and standard deviation (SD). Other categorical data are presented by number and percentage. The duration of the overall survival (OS) was defined as the time interval between the day of diagnosis and the day of death from any cause or the last day the patient was known to be alive. The duration of disease-free survival (DFS) was defined at the time interval between the diagnostic day and the day of locoregional recurrence or distant metastasis. The Kaplan-Meier estimates for survival curves were determined, and the difference between the survival curves was tested using the log-rank test. All statistical assessments were two-sided and p-values equal to or less than 0.05 were considered significant. Statistical analyses were performed using SPSS 15.0 statistics software (SPSS Inc., Chicago, Illinois, USA).

## Results

The patient characteristics and distribution of tumor status according to UFUR administration are listed Table 1. The 245 enrolled patients included 113 in the UFUR group and 132 in the comparison group. There were 125 men and 123 women, and their distribution in the two groups was not significantly different (p = 0.798). The mean age was 69.7 years in the UFUR group and 68.9 in the comparison group (p = 0.651). Their clinical characteristics including stage (IIIA vs. IIIB vs. IIIC), histological grade, lymphovascular space invasion, and tumor location were also not significantly different between the two groups.

**Table 1:** Patient characteristics.

	Comparison (n = 132)		UFER (n = 113)		
	n	%	n	%	p-value
Stage					0.311
IIIA	8	6.06	11	9.73	
IIIB	91	68.94	68	60.18	
IIIC	33	25	34	30.09	
Gender					0.798
female	64	48.48	57	50.44	
male	68	51.52	56	49.56	
Grade					0.559
1	5	3.79	3	2.65	
2	109	82.58	97	85.84	
3	18	13.64	12	10.62	
LVSI					0.897
-	76	57.58	66	58.93	
+	56	42.42	46	41.07	
Location					0.396
Ascending	44	33.33	26	23.01	
Transverse	28	21.21	19	16.81	
Descending	15	11.36	17	15.04	
Sigmoid	32	24.24	42	37.17	
Rectum	13	9.85	9	7.96	
Age					0.651
mean, sd	68.90	13.47	69.70	14.04	
Median(range)	68	35–103	71	39–94	

*Abbreviations*: LVSI = lymphvascular space invasion; sd = standard deviation.

**Fig 1:**
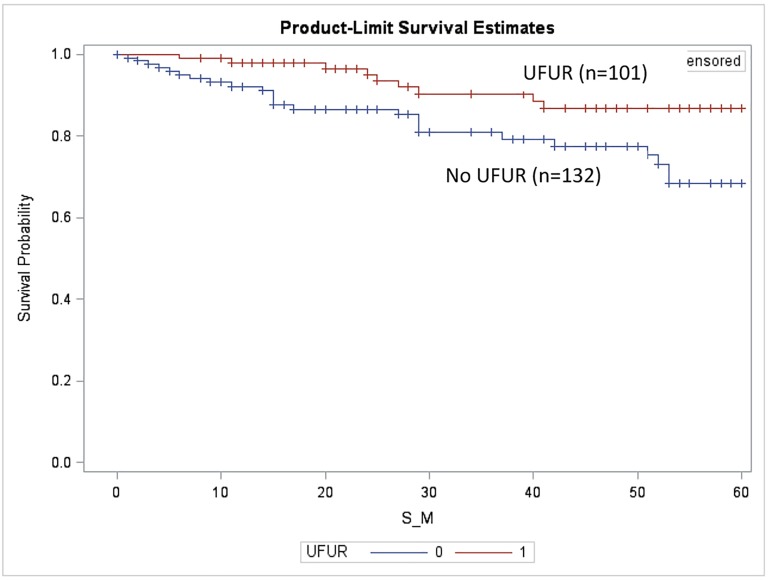
Overall survival of the UFUR group vs. the comparison group. 5-year OS: 86.8% vs. 68.5%, p = 0.01. UFUR: tegafur-uracil.

**Fig 2:**
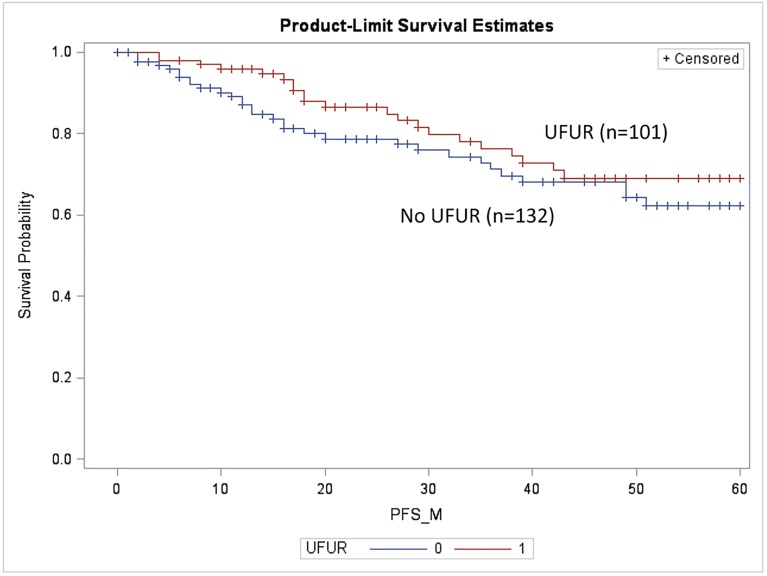
Disease-free survival of the UFUR group vs. the comparison group. 5-year DFS: 69.1% vs. 62.3%, p = 0.21. UFUR: tegafur-uracil.

The median follow-up period was 44.5 months. We compared DFS and OS in patients treated with UFUR for at least 3 months (UFUR group) with those who never received UFUR (comparison group). The OS of the UFUR group was significantly better than that of the comparison group (5-year OS: 86.8% vs. 68.5%, p = 0.01, Fig 1). There was no statistically significant difference in DFS between the two groups (5-year DFS: 69.1% vs. 62.3%, p = 0.21, Fig 2).

**Fig 3:**
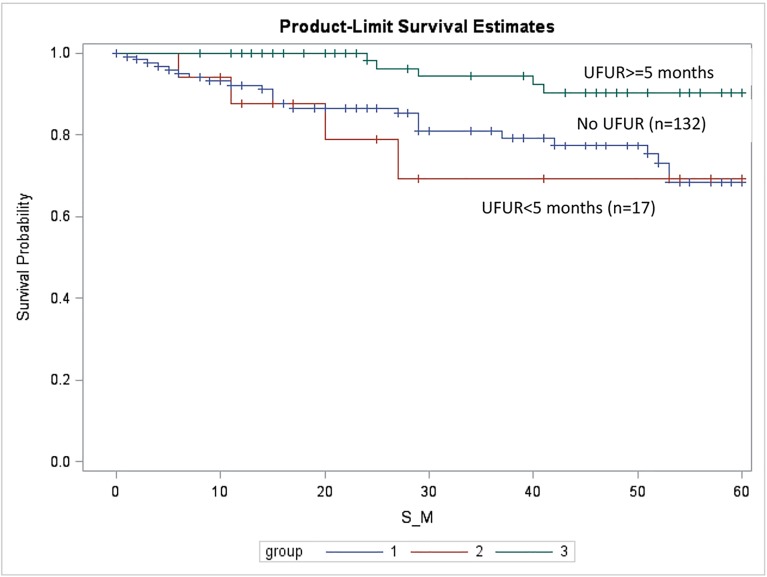
Overall survival (OS) of the comparison, UFUR <5 months, and UFUR ≥5 months groups. The 5-year OS rates of the comparison, UFUR <5 months, and UFUR ≥5 months groups were 68.5%, 69.2%, and 90.3%, respectively. UFUR: tegafur-uracil.

In order to examine the impact of the duration of UFUR therapy on outcome, we further divided the UFUR group into UFUR ≥5 months and UFUR <5 months subgroups. The 5-year OS rates of the comparison, UFUR <5 months, and UFUR ≥5 months groups were 68.5%, 69.2%, and 90.3%, respectively (Fig 3). Patients who received UFUR therapy ≥5 months had a significantly higher survival rate than those who received UFUR therapy <5 months or who received no UFUR (comparison group). (Comparison vs. UFUR ≥5 months, p = 0.002; UFUR <5 months vs. UFUR ≥5 months, p = 0.006). However, there was no difference between the survival rates of the comparison and UFUR <5 months groups (p = 0.700).

**Fig 4:**
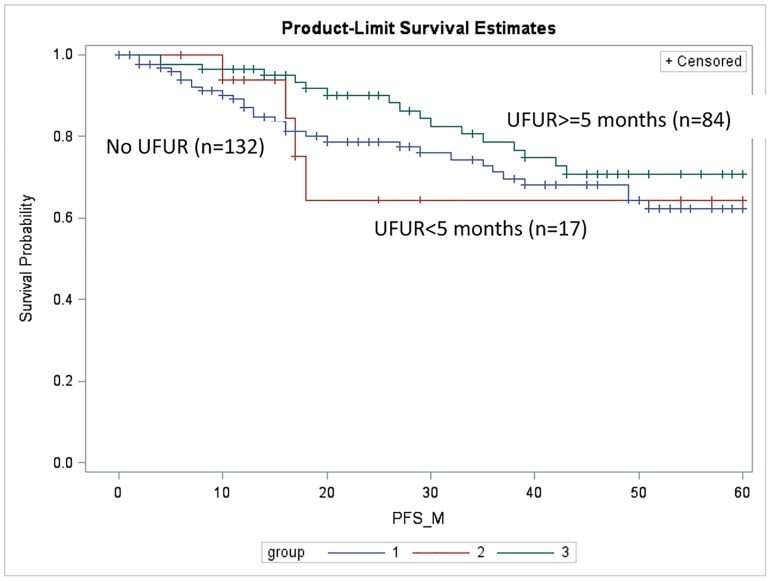
Disease-free survival (DFS) of the comparison, UFUR <5 months, and UFUR ≥5 months groups. The 5-year DFS rates of the comparison, UFUR <5 months, and UFUR ≥5 months groups were 62.3%, 64.3%, and 70.7%, respectively. UFUR: tegafur-uracil.

The 5-year DFS rates of the comparison, UFUR <5 months, and UFUR ≥5 months groups were 62.3%, 64.3%, and 70.7%, respectively (Fig 4). They were not significantly different in individual comparisons (comparison vs. UFUR <5 months, p = 0.95; comparison vs. UFUR ≥5 months, p = 0.15; and UFUR <5 months vs. UFUR ≥5 months, p = 0.23).

The treatment-related side effects were summarized in Table 2. In general, oral UFUR was well-tolerated and there were no grade 4 or 5 toxicities. Compared with the comparison group, the incidence of anorexia was statistically higher in the UFUR group (p = 0.01). There were 7 patients dropped out due to treated-related side effects (1 grade 3 leukopenia, 1 grade 3 thrombocytopenia, 2 grade 3 anemia, 2 grade 2 mucositis, 1 grade 2 nausea).

**Table 2:** Adverse effects of UFUR group vs comparison group.

	UFUR group	Comparison group	
	No. of Patients (%)	No. of Patients (%)	p-value
Leukopenia			
Any	21 (18.6)	17(12.9)	NS
Grade 3	1(0.8)	0(0)	NS
Neutropenia			
Any	19(16.8)	14(10.6)	NS
Grade 3	0(0)	0(0)	NS
Thrombocytopenia			
Any	22(19.5)	18(13.6)	NS
Grade 3	1 (0.8)	1(0.8)	NS
Anemia			
Any	29 (35.4)	36(27.3)	NS
Grade 3	13(11.5)	10(7.6)	NS
Anorexia			
Any	30 (26.5)	14(10.6)	0.01
Grade 3	0(0)	0(0)	NS
Diarrhea			
Any	15(13.2)	10(7.6)	NS
Grade 3	0 (0)	0(0)	NS
Mucositis			
Any	17 (15.0)	14(10.6)	NS
Grade 3	0(0)	0(0)	NS
Nausea/vomiting			
Any	20(17.6)	12(9.1)	NS
Grade 3	0(0)	0(0)	NS

There is no grade 4 or 5 toxicity.

## Discussion

In this study, we found that adding oral tegafur-uracil as metronomic chemotherapy for patients with stage III colon cancer increases OS. Using UFUR for more than 5 months resulted in a better survival outcome, with a 5-year OS rate of 90.3%.

The UFUR group had higher 5-year DFS, but not reached statistical difference (69.1% vs. 62.3%, p = 0.21). However, the UFUR group had significantly better 5-year OS (86.8% vs. 68.5%, p = 0.01). One possible explanation is the distinct inhibitory effect of metronomic UFUR therapy on tumor metastasis. Thus, the patients with metronomic UFUR might have lower disease burden when cancer relapse. This is compatible with the finding reported Shaked et al.[18] They found oral daily metronomic chemotherapy, using cyclophosphamide and UFT, had high anti-metastatic effect. They also found that low dose metronomic chemotherapy, compared with maximum tolerated dose, had better therapeutic advantage on advanced metastatic disease.

The attractiveness of a continuous administration of low-dose chemotherapy might lie in its anti-angiogenic effects. Some in vitro studies showed that the endothelial cells of newly forming capillaries were highly sensitive to low dose continuous chemotherapy. Some metronomic chemotherapy could induce sustained suppression of circulating endothelial progenitor cells and increase the levels of the endogenous angiogenesis inhibitor thrombospondin 1, both of which could suppress neovascularization.[19] Also, several studies showed the antiproliferative, migration-inhibitory and cytotoxic effects of very low concentrations of chemotherapeutic drugs on various cell types, including fibroblasts, lymphocytes, tumour cells, epithelial cells from various tissues, and microvascular or macrovascular endothelial cells. Potential underlying mechanisms were also proposed, such as restoration of anticancer immune response and induction of cancer dormancy.[20]

Although maximum tolerated chemotherapy kills most cancer cells, it may promote the growth of residual tumor cells through stromal reaction and interaction of tumor microenviroment. This may lead to cancer local relapse and distant metastasis. A recent study conducted by Chan et al showed that resistance to treatment is caused by expansion of stem-like tumor-initiating cells. The maximum tolerated chemotherapy enhanced STAT-1 and NF-κB activity in carcinoma-associated fibroblasts, leading to secretion of ELR motif–positive (ELR+) chemokines, which signal through CXCR-2 on carcinoma cells to trigger them to converse to tumor-initiating cells. Also, they promoted their ability of invasiveness. However, low dose continue chemotherapy could prevent stromal ELR+ chemokine paracrine signaling. This was an interesting finding and probably another therapeutic mechanism of metronomic chemotherapy.[21]

The Dutch Colorectal Cancer Group (DCCG) investigated the benefit of maintenance treatments in patients with metastatic colorectal cancer (CAIRO3 phase 3 randomized controlled trial). Patients were randomly assigned to either maintenance group (continuous low-dose capecitabine with combination of bevacizumab) or observation group after six 3-weekly cycles of capecitabine, oxaliplatin, and bevacizumab (CAPOX-B) with stable disease or better. The primary endpoint of median progression-free survival was significantly improved in patients on maintenance treatment (11.7 vs. 8.5 months; HR = 0.67, 95% CI 0·56–0·81, p<0·0001). Although patients with maintenance treatment experiences higher grade 3–4 adverse events, the global quality of life did not deteriorate during maintenance treatment and was clinically not different between treatment groups. [22]

In Japan, oral tegafur-uracil/leucovorin is widely used as a standard adjuvant chemotherapy regimen for colon cancer. Its efficacy and safety has been investigated in definitive treatment or palliative care. Phase II studies of advanced or metastatic colon cancer treated with oral tegafur-uracil/leucovorin achieved adequate disease control rates and acceptable tolerability.[23–25] In phase III studies, oral tegafur-uracil/leucovorin provided a safer, more convenient alternative to a standard bolus, such as 5-FU/LV for advanced or metastatic colon cancer, resulting in similar survival outcomes.[26,27]

Hone et al. conducted a retrospective study to evaluate the impact on outcome of adding oral tegafur-uracil after intravenous 5-FU/leucovorin as adjuvant chemotherapy for stage II and III colon cancer. They found that there is no significant difference in DFS for patients with stage II disease. On the contrary, for stage III patients, 3-year DFS rates were 80.0% and 60.7% in the 5-FU+ tegafur-uracil and 5-FU only groups, respectively (hazard ratio = 0.32; p = 0.01; 95% confidence interval = 0.13–0.76). However, some limitations existed. Nowadays, 5-FU/leucovorin is not the standard regimen for stage III colon cancer. FOLFOX is superior to 5-FU/leucovorin.[28,29] Moreover, the number of patients is relatively small, with 101 in the 5-FU+ tegafur-uracil group and 47 in the 5-FU only group. For stage III patients, there were only 45 in the 5-FU+ tegafur-uracil group and 21 in the 5-FU only group. In our study, we focused on patients with stage III disease, and they were treated with adjuvant chemotherapy with a FOLFOX regimen, which is the current standard treatment.

The optimal duration of tegafur-uracil use is unclear. The present study showed that patients who underwent UFUR therapy for ≥5 months had a significantly higher survival rate than those who received UFUR for <5 months or who received no UFUR. In a phase III randomized trial conducted in Japan (JFMC33-0502), they compared treatment results of stage IIB/III colon cancer patients after adjuvant chemotherapy with UFT/LV for 6 months or 18 months.[30] The DFS and OS rates were very similar between the two groups. The 5-year DFS rate was 68.8% in the 6 months group and 68.9% in the 18 months group. The 5-year OS was 84.9% in the 6 months group and 84.5 in the 18 months group. They suggested that a treatment duration of 6 months is sufficient for stage IIB/III colon cancer.

There were some limitations inherent to the present study. First, this was a retrospective study at a single institute. The evidence-base and statistical methods used in such retrospective studies are inferior to those from prospectively randomized trials. Thus, the prognostic impact of the metronomic use of UFUR on patients with stage III colon cancer needs to be confirmed by large, prospective studies. Second, we did not report the side effects because it was likely that some of the toxicities could not be captured from a review of the patient charts. Nonetheless, previous studies showed that toxicity associated with oral UFUR is relatively uncommon and mild.[7,21]

## Conclusion

In conclusion, adding UFUR to a FOLFOX regimen was found to significantly improve OS in patients with stage III colon cancer. Using UFUR for more than 5 months resulted in better survival outcomes than use for less than 5 months. The authors carefully suggest that UFUR as a maintenance therapy following FOLFOX regimen could be another option for the treatment of stage III colon cancer patients. However, the establishment of UFUR for routine use in stage III colon cancer awaits further prospective large-scale studies.
